# Prophylactic Antibiotic Therapy in Cleft Surgery—A Scoping Review

**DOI:** 10.3390/dj14010056

**Published:** 2026-01-15

**Authors:** Margareta Budner, Marcelina Podleśna, Aleksandra Domańska, Natalia Pijas, Katarzyna Zyska, Daniel Wiśniewski, Klaudiusz Garbacki, Grzegorz Wilhelm, Kamil Torres, Jerzy Strużyna, Agnieszka Surowiecka

**Affiliations:** 1 Department of Jaw Orthopedics, Medical University of Lublin, 20-059 Lublin, Poland; 2 Department of Plastic, Reconstructive and Microsurgery, Medical University of Lublin, 20-059 Lublin, Poland; 3 Doctoral School, Medical University of Lublin, 20-059 Lublin, Poland; 4 Międzyleski Specialist Hospital in Warsaw, 04-749 Warsaw, Poland; aleksandradomanska0206@gmail.com; 5 Infant Jesus Clinical Hospital University Clinical Center, 02-005 Warsaw, Poland; 6 Student Scientific Club of Plastic, Reconstructive Surgery and Aesthetic Medicine, Medical University of Lublin, 20-059 Lublin, Poland; 7 East Center of Burns Treatment and Reconstructive Surgery, Medical University of Lublin, 20-059 Lublin, Poland

**Keywords:** cleft lip, cleft palate, antibiotic prophylaxis, therapeutics, perioperative care, oral hygiene, drug resistance, microbial

## Abstract

**Background/Objectives:** Cleft lip and/or palate are common craniofacial anomalies whose surgical repair is classified as clean-contaminated and may be complicated by surgical site infection or palatal fistula. Despite widespread perioperative antibiotic use, there are no standardized, evidence-based recommendations, and rising antimicrobial resistance underlines the need for rational prescribing. This systematic scoping review aimed to map current evidence on prophylactic antibiotic therapy and related perioperative measures in cleft surgery. **Methods:** A scoping review was conducted using the Arksey and O’Malley framework and reported in line with PRISMA 2020. PubMed, Mendeley and Google Scholar were searched (January 2015–10 February 2025) for English-language retrospective studies, clinical trials, survey studies and systematic reviews concerning prophylactic antibiotics, bone grafting procedures, mouthwash use or oral microbiota in patients undergoing cleft lip and/or palate surgery. Six reviewers independently screened records; two experienced clinicians extracted data on study characteristics, antimicrobial regimens and infectious or microbiological outcomes. Given heterogeneity and the scoping aim, no formal risk-of-bias assessment or meta-analysis was performed. **Results:** A total of 40 studies met the inclusion criteria, including 21 original research articles. Considerable variation in antibiotic choice, timing and duration was observed, with no clear superiority of any regimen. Single-dose perioperative prophylaxis appeared non-inferior to prolonged courses in several settings. Oral microbiota studies highlighted colonization by resistant and opportunistic pathogens in cleft patients. **Conclusions:** Current evidence supports individualized, often short-course perioperative antibiotic strategies rather than routine prolonged therapy. High-quality randomized and microbiological studies are required to develop standardized, resistance-conscious guidelines.

## 1. Introduction

Cleft defects are a category of congenital anomalies that primarily affect the craniofacial region, encompassing the upper lip, the alveolar process, and the soft or hard palate. The average incidence of births presenting with cleft lip, with or without cleft palate, is 9.02 per 10,000 births in Europe, while cleft palate is 6.02 per 10,000 births [1]. The association between the presence of orofacial clefts and an impaired quality of life has been demonstrated. It is a well-documented fact that infants born with these defects are often afflicted with difficulties with feeding and aspiration of food, as well as recurrent otitis media. Furthermore, newborns with orofacial clefts demonstrate a higher rate of failure in hearing screening tests. The development of speech problems in late childhood has also been observed [2].

The classification of cleft defects is predicated on three principal factors: their location, the degree of lip involvement, and the presence or absence of a cleft palate. The classification of cleft palates can be categorized into the following types: unilateral, bilateral, midline, and complete and incomplete, depending on the degree of nasal involvement. Furthermore, cleft defects can involve either the lip or the palate, or both the palate and the lip [2]. Orofacial clefts (OFCs) are most often non-syndromic birth defects; however, a small number of cases may be components of syndromes or genetic defects. Syndromic defects are frequently the consequence of genetic or chromosomal abnormalities, or teratogenic factors, and they co-occur with other congenital abnormalities [3]. Non-syndromic OFCs have been associated with numerous risk factors, including gender, origin and maternal health status [4].

There are no guidelines that specify the treatment of cleft defects. Also, the surgical techniques used vary from one center to another. Furthermore, the surgical techniques used vary from one center to another. According to recommendations, surgical repair of cleft lip should be performed within the first year of life, and cleft palate within the first 18 months. Untreated defects have been linked to increased morbidity and mortality among patients [5,6]. An unrepaired cleft lip and palate is believed to increase the risk of colonization with pathogenic bacteria of the oral cavity and nasopharynx. Cleft surgery is categorized as clean-contaminated, and postoperative complications are predominantly associated with perioperative pathogens [7]. The most common complication of the procedure is surgical site infection (SSI) and the occurrence of palatal fistula. SSIs can lead to bacteremia, prolong hospitalization, and require reoperation. The most prevalent pathogens responsible for such infections are *Staphylococcus aureus* and B-hemolytic Streptococcus. There are no single guidelines for the use of prophylactic antibiotic therapy, as the incidence of complications is low [8]. In most cases, a single dose of a broad-spectrum antibiotic is administered to prevent serious local and systemic consequences associated with rare pathogens [9]. The Surgical Infection Society justifies the use of prophylactic antibiotic therapy if the benefits outweigh the risks. There is evidence supporting the efficacy of postoperative antibiotic therapy in reducing the risk of complications. This recommendation is of particular relevance in low-income nations, where the availability of diagnostic tools and treatment options for complications such as surgical site infections (SSIs) and palatal fistulas is often limited [10].

The objective of this systematic scoping review was to systematically map and synthesize the available evidence on prophylactic antibiotic therapy, perioperative antiseptic strategies and oral microbiota in cleft lip and/or palate surgery, and to identify remaining knowledge gaps relevant for future research and guideline development.

## 2. Materials and Methods

The review was conducted based on the Arksey and O’Malley framework for scoping reviews [11]. In addition, the reporting of this review followed the PRISMA 2020 statement (Table S1: PRISMA checklist 2020) for systematic reviews, where applicable.

### 2.1. Data Source and Search Strategy

6 independent researchers (MP, AD, NP, KZ, DW, KG) screened the articles in medical databases (PubMed, Mendeley, Google Scholar). The results were filtered by year of publication. A systematic electronic search of these databases was performed from January 2015 to 10 February 2025. The results were filtered by year of publication. Only full- text articles meeting the inclusion criteria were taken into consideration. Preliminary search in broader databases did not provide any additional, non-duplicating and scientifically significant publications about our topic. Almost all clinical studies on prophylactic antibiotic therapy in patients with cleft palate and lip defects were found in either PubMed, Mendeley or Google Scholar. Our work is a scoping review, not a systematic one; therefore, we decided to restrict our search strategy to the 3 mentioned databases. The papers were analyzed, and duplicates were removed. No automation tools were used at any stage of the search or de-duplication process. In addition, the reference lists of all included articles and of relevant reviews were screened manually to identify further eligible studies.

### 2.2. Inclusion Criteria

Papers from the period 2015–2025 were included in the reviewing process. Additionally, the authors decided to include a few older articles from 1990 to 2013, because of their scientific relevance and contribution to the topic. In the course of our preliminary research, we established that a number of older articles had been repeatedly cited in new studies, thereby establishing a foundation for the current state of knowledge. A thorough analysis was conducted to assess the continued relevance of these documents in 2025 and their unique contribution to the field. Since this work is not a systematic review, we decided to make an exception for these few significant works. Therefore, in citations, despite the fact that our search was limited to works from 2015 to 2025, we also decided to mention these historically influential studies in our citations section. The inclusion criteria comprised studies written in English, retrospective studies, clinical trials, survey studies and systematic reviews. Full- text articles were discussed and included in the main text. In addition, articles on oral flora and mouthwash solutions were included. Given that the operation is performed within the oral cavity, the bacterial flora plays a crucial role in postoperative healing. Mouthwashes are important because they can reduce microbiological contamination and, with that, the risk of infection. Furthermore, they can promote wound healing. In addition to these benefits, they can be considered a valuable supplement or alternative to systemic antibiotics. The inclusion of these studies therefore helps to create a more comprehensive picture of perioperative management for cleft lip and palate surgery. The terms used to find articles were created by combining terms related to cleft lip and palate and antibiotic prophylaxis. The search strategy involved a combination of keywords such as “cleft lip”, “cleft palate”, “orofacial clefts”, “microbiota cleft palate”, “microflora cleft palate”, “oral flora cleft palate”, “cleft lip repair”, “cleft palate repair”, “palatoplasty”, “graft”, “bone graft”, “bone tissue transplantation”, “autologous bone graft”, “allogeneic bone graft”, “mouthwash”, “mouthrinse”, “antibiotic prophylaxis”, “perioperative antibiotic”, “infection prevention” and “surgical site infection.” Studies were included if they reported at least one of the following: composition of the oral microbiota or colonization with specific pathogens in patients with cleft lip and/or palate; use of perioperative antibiotic prophylaxis in cleft lip and/or palate surgery; antibiotic use in procedures involving bone tissue transplantation in cleft-related defects; or use of mouthwash or other antiseptic solutions in the perioperative management of these patients. For the purposes of synthesis, eligible studies were subsequently grouped into four prespecified thematic domains: oral microbiota and pathogens in cleft-related conditions, antibiotic prophylaxis in isolated cleft defects, antibiotic prophylaxis in cleft lip and palate defects, and perioperative management in procedures involving bone grafts and antiseptic mouthwash solutions.

### 2.3. Exclusion Criteria

During the selection of scientific sources for this article, publications not directly related to the topic of oral microflora and antibiotic therapy in cleft lip and palate defects were initially excluded. Articles published in languages other than English were also excluded due to the adopted language limitations and the impossibility of a detailed analysis of the content of such works. The review also excluded publications to which there was no access in full text, which made it impossible to assess their methodology and results. In the case of duplicate publications or several reports on the same study, only the most complete version or the one containing the most detailed data was included. Conference abstracts, editorials, narrative reviews without original data and single-case reports were also excluded.

### 2.4. Data Extraction and Synthesis

Two independent researchers (MB, AS) independently reviewed the included studies, extracted the data, and discussed the outcomes. Both researchers have extensive clinical experience and academic backgrounds related to surgical treatment, particularly in the field of plastic and reconstructive surgery. The work was supervised and directed by MB whose experience in the field of cleft defect surgery dates back to 2008. For each included study, we extracted the following variables, where available: study design, country and setting, study period, sample size, age and sex distribution, type and extent of cleft, type of surgical procedure, details of the perioperative antibiotic regimen (agent, dose, timing and duration), use of mouthwash or other antiseptic measures, type of bone grafting procedure (if applicable), length of follow-up and all reported outcomes related to infection (surgical site infection, palatal fistula, graft failure, wound dehiscence, bacteremia) and oral microbiota composition. When multiple outcome measures or time points were reported, all results compatible with our predefined outcome domains were extracted. Any discrepancies in data extraction were resolved by discussion and, when necessary, by consultation with a supervisor. Study authors were not contacted for additional or missing data; unclear or missing information was recorded as “not stated” and described narratively. Given the scoping nature of this review, we did not perform a formal risk-of-bias assessment of individual studies and did not apply a GRADE or other framework to assess certainty (confidence) in the body of evidence. Likewise, no quantitative meta-analysis, subgroup analysis, meta-regression or sensitivity analysis was conducted. Instead, we used a descriptive synthesis approach, tabulating key study characteristics and results in Table 1 and organizing the narrative synthesis into four thematic sections. Heterogeneity between studies was explored qualitatively by comparing study designs, populations, interventions and outcome definitions across these themes.

## 3. Results

**Figure 1:**
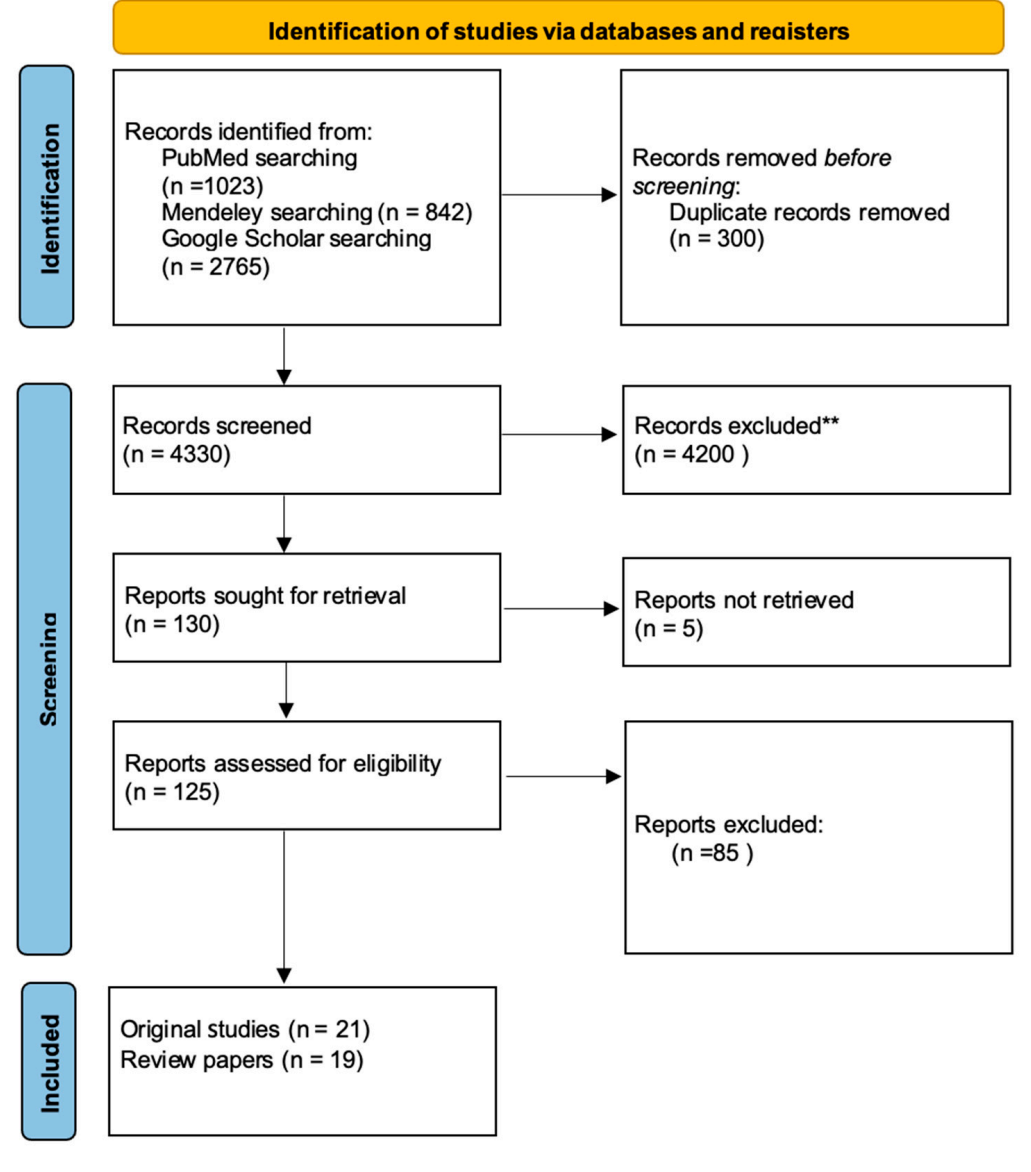
Study selection process.

A total of 40 papers were identified with the searching strategy. The records came from PubMed, Mendeley and Google Scholar. It is important to note that only articles that met the inclusion criteria were taken into consideration. Employing the keywords previously specified within the inclusion criteria, a total of 4330 works were identified in the databases. Figure 1 is a graphical presentation of the screening process according to the PRISMA guidelines [Figure 1]. The accompanying flow diagram illustrates the number of records that have been identified, screened, and assessed for eligibility. The final selection comprised 19 review papers and 21 original research articles. While the incorporation of all 40 papers was intended to provide a comprehensive overview of the topic, the detailed synthesis of clinical and microbiological outcomes was based on the 21 original studies in order to focus on primary data. The original works published between 2008 and 2025 addressed the following subjects: the prevention of antibiotic-resistant infections in cleft lip and palate defects; mouthwash solutions used in perioperative management; and the composition of the oral flora in patients with the aforementioned defects. The study groups included 27 to 7160 participants, depending on the nature of the study. Research included studies from various countries, with the largest contributions from the USA, the United Kingdom, India, and Germany. Additionally, we incorporated 2 studies each from the Netherlands and Sweden, and 1 study from each of the following countries: Brazil, Poland, Colombia, Australia, Belgium, Italy, South Africa, Iran and Nigeria. Table 1 presents a summary of the results of the original studies chosen for the work [Table 1]. A review of the literature on the composition of the oral microflora in patients with cleft defects was conducted, resulting in the selection of six papers that provide a detailed and reliable account of the aforementioned issue. A total of 10 studies were included in order to present various perspectives on the problem of perioperative antibiotic therapy after palatoplasty, and 9 works relating to co-occurring cleft lip and palate defects. A total of 7 studies evaluating the effectiveness of various prophylactic antibiotic protocols in bone tissue transplantation were included in the discussion. In the field of oral surgery, particularly concerning cleft lip and palate treatments, five important studies were chosen. As no formal risk-of-bias or reporting bias assessment was undertaken, the results presented below should be interpreted as a map of the existing evidence rather than as pooled estimates of treatment effects.

## 4. Discussion

### 4.1. Oral Pathogens in Concomitant Cleft Defects

The analysis of relevant studies indicates significant differences in the oral microbiota composition of patients with cleft palate or cleft lip and palate compared to healthy individuals. Patients with these congenital anomalies have been found to be colonized by specific pathogens, including *Moraxella catarrhalis*, *Staphylococcus aureus*, *Enterobacter cloacae*, *Klebsiella pneumoniae*, and *Klebsiella oxytoca*, which are atypical for individuals without clefts. These pathogens may have a significant impact on postoperative wound healing, particularly concerning oronasal fistula formation, one of the most common complications following reconstructive procedures, as well as a variety of other health issues, including an increased risk of caries, periodontal disease, gingivitis, pneumonia and bronchiolitis [30,31,32,33]. Special attention should be paid to β-lactamase-producing bacteria such as *M. catarrhalis* and methicillin-resistant *Staphylococcus aureus* (MRSA). The presence of these pathogens may explain the clinical observation of ineffective routine antibiotic prophylaxis using standard β-lactams, suggesting a necessity to modify antibiotic protocols towards broader-spectrum antibiotics such as amoxicillin/clavulanate [12,30]. Moreover, these findings emphasize the necessity for enhanced aseptic measures and optimized infection control measures during hospitalization for cleft patients, particularly given the risk of acquiring MRSA strains during repeated surgical procedures. Furthermore, the analysis demonstrates a reduction in the colonization of beneficial bacteria, including *Streptococcus mitis*, *S. salivarius*, *S. gordonii*, and *Fusobacterium nucleatum*, which may potentially result in dysbiosis and an increased risk of caries and periodontal diseases [31]. Elevated levels of *Streptococcus mutans* and *Lactobacillus* spp. were also noted, with a direct association to an increased risk of dental caries. A significant pathogenic factor is the heightened colonization by *Candida* spp., which may contribute to mucosal infections [33,34]. However, it must be emphasized that literature data are somewhat inconsistent regarding specific aspects of the oral microbiome in cleft patients, indicating the need for further research utilizing advanced microbiological techniques. Such studies could allow a more detailed analysis and understanding of the complex interactions between microorganisms and the host, facilitating the development of more effective preventive and therapeutic strategies. From a clinical perspective, it is crucial to consider the impact of orthodontic and surgical interventions on microbiological dynamics. These treatments may further increase the risk of infectious complications in patients with cleft palates.

### 4.2. Antibiotic Prevention in Isolated Defects

On the one hand, Aznar et al. demonstrated that antibiotics can reduce the risk of complications, especially early infections. Nevertheless, the authors also emphasize the necessity for long-term patient monitoring, due to the potential for delayed fistula formation [29]. This finding indicates that complications may be underestimated during brief follow-up periods. On the other hand, Narayan et al. found that postoperative antibiotic therapy was not necessary [18]. It is noteworthy that the study incorporated a small sample of patients and the follow-up period was only three months, which may have affected the conclusions. Jodeh et al.’s analysis also revealed an absence of correlation between the type of antibiotic administered and the time of administration with the risk of fistula during palatoplasty [20]. These findings imply that the routine utilization of antibiotics, particularly in the preoperative period, may not provide substantial clinical benefits. Based on the analysis, Davies et al. reported appreciable differences in the incidence of fistula according to the duration of antibiotic therapy or the type of drug used [19]. Rottgers et al. proposed a compromise approach and recommended the administration of an antibiotic in the perioperative period, particularly a single dose of ampicillin with sulbactam (50 mg/kg) prior to the incision of the skin. At the same time, however, they questioned the point of postoperative therapy [9]. The study by Sitzman et al. revealed significant variability in postoperative antibiotic use among surgeons performing cleft palate repair in the United States. Only 45% of surgeons reported using antibiotics, with notable differences in the choice of agents and duration of administration [22]. Moreover, the use of an aforementioned antibiotic oral pack for 5 days in combination with antibiotics demonstrated efficacy in reducing the incidence of fistula [21]. Perioperative antibiotic prophylaxis in cleft lip surgery is often restricted to a single dosage given during induction [23,24,25]. As demonstrated in the reviewed studies, infection and wound dehiscence rates remain at relatively low levels. In both isolated cleft lip and cleft palate procedures, the duration of antibiotic use did not exceed seven days. In the studies analyzed, the most commonly used antibiotics in the treatment of cleft palate were: cefazolin, co-amoxiclav, amoxicillin, cefuroxime, cefotaxime, clindamycin, penicillins (including phenoxymethylpenicillin, flucloxacillin alone and in combination with ampicillin) and metronidazole (in one of the combination therapies). The preceding studies indicate that cefuroxime, co-amoxiclav and flucloxacillin were used in the treatment of cleft lip. The accumulated data clearly indicate the need for further studies of high methodological quality, especially randomized clinical trials with an adequate follow-up period to assess the real efficacy and validity of postoperative antibiotic therapy.

### 4.3. Antibiotic Prophylaxis in Cleft Lip and Palate Defect

Congenital defects, including cleft lip and palate, present a substantial clinical and epidemiological challenge, mandating early surgical intervention. The controversy surrounding the issue of antibiotic prophylaxis is a consequence of the location of the procedure within the oral cavity, which is naturally colonized by bacteria. The present text discusses the current data on the effectiveness of perioperative antibiotic therapy and its impact on postoperative complications in this group of patients, based on nine scientific studies addressing cleft lip and palate defects. Corrective surgeries are performed in the oral cavity, which is naturally colonized by bacteria. For this reason, these procedures are classified as clean-contaminated wounds (class II) [10]. There is no consensus regarding the effectiveness of prophylactic antibiotic administration following such surgeries. However, a meta-analysis by Aryan et al. suggests that the administration of a single dose of antibiotics prior to the procedure can significantly reduce the risk of surgical site infections (SSIs) [35,36]. In this context, the use of ampicillin with sulbactam at a dose of 50 mg/kg is recommended in newborns and children [35,36]. In a clinical trial of cleft palate surgery, a five-day course of amoxicillin (50 mg/kg/day) was utilized, leading to a decline in the incidence of fistulas from 17.1% to 10.7% [29]. Although the results were favorable, the differences did not reach statistical significance—similarly to the reduction in the incidence of other early complications (13.8% vs. 8.7%, *p* = 0.175) [29]. However, a large retrospective study of over 3100 patients revealed no significant differences in the incidence of wound infections between patients who received postoperative antibiotics and those who did not [23]. Furthermore, attention was drawn to the risk of postoperative bacteremia—in 53% of patients, the presence of bacteria in the blood persisted for up to 15 min after surgery [32,37,38]. The occurrence of this phenomenon was found to be more frequent in cases of lip procedures (40.9%) in comparison to palate procedures (33.3%) [32,37,38]. This information is of particular importance in patients with heart defects, who may be at greater risk of systemic complications resulting from bacteremia. From a practical standpoint, antibiotics are more frequently employed in cleft lip surgery than in cleft palate surgery [8,29]. Nevertheless, the data supporting this approach are limited. Current recommendations concentrate primarily on perioperative prophylaxis, while the use of postoperative, long-term antibiotics should be adequately justified and individualized. In the context of the global problem of increasing antibiotic resistance, a rational approach to antibiotic therapy is gaining importance. Decisions regarding its use should take into account a number of factors, including the degree of cleft, the patient’s age, possible comorbidities, socioeconomic conditions, and access to specialist care. Large, multicenter studies are still required to more precisely assess the effectiveness and validity of different antibiotic regimens in this group of patients [32].

### 4.4. Antibiotic Prophylaxis in Defects Requiring Bone Tissue Transplantation

Antibiotic prophylaxis remains a key component of perioperative care in procedures involving bone grafting for cleft-related defects. The purpose of this prophylaxis is to lower the risk of postoperative infections and improve surgical outcomes. Despite its clinical significance, there is still no standardized, evidence-based guideline on the optimal antibiotic course for such situations. The present discussion is based on an analysis of seven studies evaluating the effectiveness of various prophylactic antibiotic protocols in bone tissue transplantation. However, it is important to emphasize that the majority of the included studies involved relatively small patient cohorts, which limits the generalizability of the conclusions. The current state of evidence suggests that the selection of antibiotic agents for prophylaxis may have no substantial impact on postoperative infection rates. A review of recent literature on the subject revealed a number of antibiotics that have been reported in various publications, including amoxicillin-clavulanic acid, cefuroxime, clindamycin, penicillin and penicillin G. These antibiotics appear to offer comparable protection against infection [13,14,15,16,17,36,39]. This finding indicates that in clean-contaminated surgeries, the timing and dosage of antibiotics may be more crucial than the specific agent employed. It is important to note that other significant perioperative considerations include the patient’s oral hygiene, especially in younger patients. Amoxicillin is a reasonable first-line choice for most cleft-related bone grafting procedures due to its affordability and availability [16,36]. However, more extensive surgical interventions, such as secondary alveolar bone grafting (SABG) or operations involving complex anomalies like bilateral cleft lip and palate, may require a more tailored approach and prolonged treatment. Such cases are predisposed to complications, including wound dehiscence and graft failure. In such contexts, clindamycin is often preferred for its strong activity against anaerobes [14]. An important finding identified in the reviewed studies is the non-inferiority of single-dose antibiotic prophylaxis compared to prolonged regimens. In many cases, patients who received a single dose experienced shorter hospital stays without an increased risk of postoperative infection [13,17,39]. These findings are particularly relevant in the context of growing antimicrobial resistance. In conclusion, the current evidence supports the use of simplified, single-dose preoperative prophylactic antibiotic protocols for standard cleft-related bone grafting procedures. Extended courses and broad-spectrum antibiotics should be reserved for selected high-risk patients. However, further randomized controlled trials are needed to confirm these findings and to help develop standardized prophylactic guidelines for this patient population.

### 4.5. Mouthwash Solutions Used in Perioperative Management

Following a comprehensive review of the extant literature on mouthwashes in the context of oral surgery, including cleft lip and palate procedures, five key studies were selected for further analysis. These studies form the basis for further analysis of the effectiveness of various antiseptic solutions in the perioperative period. Chlorhexidine is widely regarded as the gold standard; however, its inability to fully eradicate pathogens, in conjunction with the risk of antimicrobial resistance, has prompted the exploration of alternative solutions. It is important to note that effective mouth rinsing regimens not only contribute to reducing pathogenic oral microflora, but may also play a crucial role in minimizing the need for systemic antibiotics in the postoperative period. Chlorhexidine is widely regarded as the gold standard in perioperative antiseptic management, particularly in oral surgery, including procedures related to cleft lip and palate. In the early postoperative period, the utilization of mouthwashes containing 0.2% chlorhexidine gluconate is recommended as a means of supporting gingival health and reducing inflammation [40]. However, studies have demonstrated that chlorhexidine does not adequately eliminate pathogens from the surgical site prior to procedures involving the soft palate. This may result in an elevated risk of postoperative infections and the development of microbial resistance. These concerns have prompted the search for effective and safe alternatives that could support wound healing while also reducing the risk of selecting resistant bacterial strains [26]. Silver nanoparticle-based mouthwashes appear to be a promising area of research; in animal models, they have demonstrated antimicrobial efficacy comparable to chlorhexidine and simultaneously promoted wound healing [27]. The effectiveness of chlorhexidine solution, warm saline, and plain warm water as mouth rinses following oral surgery has also been compared. The findings indicate that all three methods were equally efficacious in limiting bacterial colonization on sutures, which may suggest the potential for more accessible options in the prevention of postoperative oral infections [28]. In a study on orthognathic surgery, povidone-iodine mouthwash was shown to be as effective as chlorhexidine in reducing bacterial load in the oral cavity. This raises the question of whether such a solution could also be used in patients undergoing cleft lip and palate surgery [41]. It is important to emphasize that data on the effectiveness and safety of different mouth rinses in the context of cleft lip and palate surgery remain very limited. However, well-designed, randomized clinical trials are lacking; that is to say, studies that could clearly identify which rinses are most effective in preventing infections and supporting healing in this patient group. In light of the elevated microbial load present within the oral cavity, the implementation of a well-informed, evidence-based mouthwash policy has the potential to substantially enhance infection control and surgical outcomes.

### 4.6. Limitations

From a methodological perspective, this review has several limitations. First, it was not registered a priori and no formal protocol was published, which may increase the risk of selective reporting. Second, because of the heterogeneity of designs, populations and outcome definitions and the scoping aim, we did not perform a formal risk-of-bias assessment, reporting bias assessment or certainty-of-evidence grading, and we did not undertake any quantitative synthesis, subgroup analysis or sensitivity analysis. Third, the restriction to English-language, full-text articles and to three electronic databases means that relevant evidence published elsewhere may have been missed. These factors should be taken into account when interpreting the findings and their implications for clinical practice.

## 5. Conclusions

The oral microbiota of cleft patients has been demonstrated to exert a substantial influence on postoperative outcomes. Infection is the most common complication, occurring not only in the perioperative period but also preceding final reconstructions of oral defects. In the contemporary era, characterized by the prevalence of multiresistant pathogens, it is imperative to implement a rational antibiotic policy. As various authors have noted, single-dose broad-spectrum antibiotics are frequently employed, with dosage adjustments being made according to the patient’s postoperative clinical status. There is an absence of consensus regarding perioperative prophylactic antibiotic therapy in cleft lip or cleft palate surgeries. The limitation of randomized multicenter studies on larger cohorts of comparable patients with follow-up is that it is not possible to establish unables stating clear guidelines. Observations of mouthwash antiseptic solutions have been promising, but no single product has sufficient data to support routine usage. It is recommended that future research place a priority on well-designed, adequately powered randomized controlled trials with standardized outcome definitions and sufficient follow-up. These should be complemented by high-quality microbiological studies, with the aim of enabling formal risk-of-bias and certainty-of-evidence assessments. The results of such research will inform evidence-based, resistance-conscious clinical guidelines.

## Figures and Tables

**Table 1:** The summary of results.

Title	Year of Publication	Country	Type of Study	Patients and Methods	Number of Participants	Middle Age	Type of Cleft	Type of Intervention	Results, Conclusions
Oral *Staphylococcus* Species and MRSA Strains in Patients with Orofacial Clefts Undergoing Surgical Rehabilitation Diagnosed by MALDI-TOF MS. [12]	2024	Brazil	original research	The study involved 59 patients from whom saliva samples were collected between hospitalizations. The samples were sent for microbiological diagnostics.	59 (17 female, 42 male patients)	mean of 10.1 ± 14.6 years old	Cleft lip palate	observation (microbiological swabs)	The study found various *Staphylococcus* species, including MRSA strains, in the saliva of cleft patients, confirming increased bacterial colonization.
Evaluating the efficacy of single-shot versus prolonged antibiotic prophylaxis in alveolar cleft osteoplasty: a retrospective cohort study. [13]	2023	Germany	original research	The study involved 83 patients divided into two groups. The study group received prophylactic antibiotic therapy for 4 days of the postoperative period, while the control group received one dose of antibiotics after surgery.	83 (46 male, 37 female patients)	mean age 12.8 years	Left-sided cleft lip and palate (49.5%), right-sided cleft lip and palate (25.3%), bilateral cleft lip and palate (24.2%), isolated cleft palate (1.1%)	observation	There was no significant difference in the rate of complications between single and prolonged antibiotic therapy, suggesting no benefit from longer treatment.
Incidence of complications in secondary alveolar bone grafting of bilateral clefts with premaxillary osteotomy: a retrospective cohort study. [14]	2020	Netherlands	original research	The study group consisted of 64 people who underwent SABG + PO surgery and were then given prophylactic antibiotics three times daily from the start of surgery and for 3 days postoperatively.	64 (26 female, 38 male patients)	mean age 11.37 years	bilateral cleft lip and palate	surgery	In the group of 64 patients, a significant correlation was found between the frequency of complications and the timing of the bone graft relative to the patient’s age at the time of surgery.
Outcome after secondary alveolar bone grafting among patients with cleft lip and palate at 16 years of age: a retrospective study. [15]	2021	Sweden	original research	The study included 91 patients with 100 cleft sites, treated with autogenous bone grafts. All patients were administered intravenous antibiotics immediately preoperatively and during the next 24 h.	91	16 years old	cleft palate- unilateral (82 patients); bilateral (9 patients)	observation	Most patients achieved stable graft integration and good functional outcomes, and complications were rare. Routine 24-h antibiotic therapy proved sufficient.
Success rate of mid-secondary alveolar cleft reconstruction using anterior iliac bone grafts: a retrospective study. [16]	2022	Belgium	original research	A retrospective cohort study was performed. A total of 124 patients were included and grouped as those primarily receiving a two-staged palatoplasty protocol and those receiving a one-staged protocol.	124 (48 female and 76 male patients)	First group average age 9.7 years, second group average age 11.5 years	73 had Veau III clefts, and 35 had Veau IV clefts; 15 patients had unilateral lip and alveolar clefts; 1 bilateral lip and alveolar clefting.	surgery	Bone reconstruction from the iliac crest showed high efficacy regardless of the palatoplasty protocol.
Single-dose versus prolonged antibiotic prophylaxis for alveolar bone grafting in cleft patients. [17]	2023	Germany	original research	The study included 109 patients divided into two groups: the research group received prophylactic antibiotic therapy for 2–11 days (median 5 days), and the control group received 1 dose of antibiotics preoperatively.	94 (57 male, 37 female patients)	Average age 11.5 years old	cleft lip and palate (84), cleft alveolus (16%)	observation	Limiting antibiotic prophylaxis to a single dose did not lead to a higher infection rate. These findings provide strong evidence in favor of minimizing antibiotic use in alveolar bone grafting procedures for patients with clefts.
Antibiotic prophylaxis for the prevention of fistulae in cleft palate repair: A quality improvement study. [18]	2025	United Kingdom	retrospective cohort study	The study involved 97 patients divided into two groups. The research group received prophylactic antibiotic therapy for 7 days after surgery, and the control group received one dose of antibiotics preoperatively.	97 (58 male, 39 female patients)	Average age 16.6 months	cleft palate: Veau 1 (11.1%), Veau 2 (57.4%), Veau 3 (20.3%), Veau 4 (11.2%)	observation	Extended antibiotic therapy did not significantly reduce the incidence of fistulas compared with a single dose. The results indicate that the duration of therapy does not affect the risk of complications.
Association of Perioperative Antibiotics with the Prevention of Postoperative Fistula after Cleft Palate Repair. [19]	2024	United Kingdom	retrospective cohort study	The analyses used 2136 surgical questionnaires completed at the time of palate repair in 1881 patients.	1881	Between 4.9 and 11.8 years old	cleft palate	observation	Prolonged antibiotic therapy did not significantly reduce the incidence of fistulas compared to a single dose. There was no evidence to suggest a difference in the incidence of fistulas between patients receiving amoxicillin plus clavulanic acid and those receiving an alternative antibiotic.
The Use of Prophylactic Antibiotics before Primary Palatoplasty Is Not Associated with Lower Fistula Rates: An Outcome Study Using the Pediatric Health Information System Database. [20]	2019	USA	retrospective cohort study	Retrospective study analyzed data from 7160 patients. Two different analysis models were performed. The first model included the patients who were given preoperative antibiotics, postoperative antibiotics and none. The second model included the patients who were given preoperative antibiotics.	7160	Mean age 11.5 years old	cleft palate	observation	The results showed that the use of pre- or postoperative antibiotic therapy did not significantly reduce the rate of fistulas as a complication of surgery.
Placement of an antibiotic oral pack on the hard palate after primary cleft palatoplasty: a randomized controlled trial into the effect on fistula rates. [21]	2018	India	retrospective cohort study	Study group consisted of two groups of 100 patients each, who underwent primary palatoplasty. Group A had an oral pack placed on the hard palate for 5 days postoperatively while group B (control) did not.	200	Between 12 and 13 months of age	non-syndromic complete unilateral cleft lip and palate with a previously repaired cleft lip	observation	The use of an antibiotic-impregnated dressing significantly reduced the incidence of fistulas.
Cleft Palate Repair Postoperative Management: Current Practices in the United States. [22]	2022	USA	survey study	A survey about postoperative management was administered to 67 cleft surgeons.	-	-	-	survey	The survey revealed significant variation in postoperative practices across the US, particularly regarding antibiotics and hygiene recommendations. Uniform guidelines are lacking.
Early Surgical Complications After Primary Cleft Lip Repair: A Report of 3108 Consecutive Cases. [23]	2015	India	retrospective cohort study	The medical records of 3108 consecutive lip repairs with 2062 follow-ups were reviewed retrospectively.	2062	Median 7 years old	cleft lip	surgery	The study showed that the incidence of wound infections can be kept relatively low even without the use of postoperative antibiotics.
Perioperative Management in Patients With Cleft Lip and Palate. [24]	2020	Germany	survey study	A survey was administered to members of 70 international cleft centers.	-	-	-	survey	An international survey revealed significant heterogeneity in perioperative protocols, including antibiotic use. Practices varied between centers and countries.
Prophylactic antibiotics and surgery for primary clefts. [8]	2008	United Kingdom	survey study	A survey was administered to 27 surgeons who were doing primary cleft lip surgery.	-	-	-	survey	The study showed a highly diverse approach among surgeons to the use of antibiotics in primary surgeries and the need to implement a standardized protocol for the implementation of prophylactic antibiotic therapy.
Current Surgical Practice for Children Born with a Cleft Lip and/or Palate in the United Kingdom. [25]	2023	United Kingdom	prospective cohort study	The study was based on 1782 surgical forms, related to 1514 patients with primary surgeries.	1514	Mean age for cheiloplasty 4.3 months; mean age for intravelar veloplasty 10.3 months	cleft lip; cleft palate	surgery	An analysis of procedures revealed significant differences in surgical techniques and approaches to prevention. Antibiotic therapy was used in 96% of cases, but the duration varied depending on the type of treatment.
Between unity and disparity: current treatment protocols for common orofacial clefts in European expert centers. [6]	2024	Netherlands	original research	A structured questionnaire was distributed across expert centers in the European Reference Network CRANIO. In total, 138 surgical protocols for CP, UCLP, and BCLP were identified.	-	-	-	-	138 different treatment protocols were identified across Europe, reflecting significant heterogeneity in practice.
A Descriptive Study of Chlorhexidine as a Disinfectant in Cleft Palate Surgery. [26]	2018	RPA	original research	Swabs were taken from the surgical site of 50 patients with cleft soft palate, before and after disinfecting and were sent for microbiological diagnostics.	50 (25 male, 26 female patients)	Mean age 7 months	soft palate cleft	observation	Chlorhexidine significantly reduced the number of microorganisms in the surgical site of the palate. The study confirmed its effectiveness as a disinfectant.
Comparison of antimicrobial and wound-healing effects of silver nanoparticle and chlorhexidine mouthwashes: an in vivo study in rabbits. [27]	2022	Iran	original research	Microbial samples were collected from 60 rabbits, and then standardized wounds were created. After surgery, the animal models were randomly divided into 4 groups. The control group did not receive any postoperative mouthwash, 1 group received 9.80 wt% silver nanoparticle mouthwash; group 2 received all the ingredients of the formulated mouthwash except for silver nanoparticles; group 3 received chlorhexidine 2.0% mouthwash.	-	-	-	-	In rabbits, rinses with silver nanoparticles and chlorhexidine reduced bacterial colonization, and silver showed additional healing benefits. Both treatments were effective.
A prospective clinical evaluation of the effects of chlorhexidine, warm saline mouth washes and microbial growth on intraoral sutures. [28]	2015	Nigeria	original research	The study group consisted of 100 patients. They were randomly divided into 3 groups. 1 group used chlorhexidine, 2 group used warm saline, control group used warm water mouth rinses.	100 (51 male, 49 female patients)	Range between 18 and 50 years	-	observation	The study showed that neither chlorhexidine nor warm saline mouth rinses had a meaningful impact on suture absorption time or the level of bacterial colonization on the sutures.
Role of Postoperative Antimicrobials in Cleft Palate Surgery: Prospective, Double-Blind, Randomized, Placebo-Controlled Clinical Study in India. [29]	2015	India	clinical trial	The study included 518 patients divided into two groups. The study group received 5 days of prophylactic antibiotic therapy postoperatively; the control group received a placebo. Both groups received a dose of antibiotics preoperatively.	518	Median 9 years old	cleft palate	observation	The rate of postoperative complications, including fistulas, was lower in the group of patients who received postoperative antibiotic therapy instead of placebo.
“Antibiotic Use in Primary Palatoplasty: A Survey of Practice Patterns, Assessment of Efficacy, and Proposed Guidelines for Use.” [9]	2016	USA	survey study + retrospective study	A survey was administered to surgeons (out of 1115, only 312 responded). Retrospective study group consisted of 311 patients who had undergone palatoplasty surgery. Two groups were studied. Group 1 received no antibiotics. Group 2 received preoperative and/or postoperative antibiotics.	311 patients	Mean age for group one was 3.1 years, and for group two, 2.9 years	cleft palate	observation	The study showed no significant differences between the groups in the incidence of fistulas and delayed healing. 85% of surgeons administered prophylactic perioperative antibiotic therapy, but there is no consensus on the length or type of antibiotic therapy used. Considering the possible complications that may occur, the researchers recommend a single preoperative dose of antibiotic.

## Data Availability

The original contributions presented in this study are included in the article. Further inquiries can be directed to the corresponding author.

## Supplementary Materials

The following supporting information can be downloaded at: https://www.mdpi.com/article/10.3390/dj14010056/s1, Table S1: PRISMA checklist 2020 [42].
